# Effect of Tumor Size on Long-Term Survival After Resection for Solitary Intrahepatic Cholangiocarcinoma

**DOI:** 10.3389/fonc.2020.559911

**Published:** 2021-01-21

**Authors:** Junjie Kong, Yukun Cao, Jiawei Chai, Xihan Liu, Cunhu Lin, Jianping Wang, Jun Liu

**Affiliations:** ^1^ Department of Liver Transplantation and Hepatobiliary Surgery, Shandong Provincial Hospital, Cheeloo College of Medicine, Shandong University, Jinan, China; ^2^ Department of Liver Transplantation and Hepatobiliary Surgery, Shandong Provincial Hospital Affiliated to Shandong First Medical University, Jinan, China; ^3^ Department of Breast and Thyroid Surgery, Shandong Maternity and Child Care Hospital, Jinan, China; ^4^ Cheeloo College of Medicine, Shandong University, Jinan, China; ^5^ Department of Pathology, Shandong Provincial Hospital Affiliated to Shandong First Medical University, Jinan, China

**Keywords:** intrahepatic cholangiocarcinoma, tumor size, solitary, resection, overall survival, SEER

## Abstract

**Background:**

The relationship between tumor size and survival in intrahepatic cholangiocarcinoma (ICC) is still controversial. This study aimed to evaluate the prognostic ability of tumor size for solitary ICC after resection and explore optimal cut-off values in different subgroups.

**Methods:**

Patients with solitary ICC who underwent liver resection from the Surveillance, Epidemiology, and End Results Program and Shandong Provincial Hospital were retrospectively analyzed. Kaplan-Meier and Cox regression analysis were used to assess the prognostic ability of tumor size. The log-rank test was used to determine the optimal cut-off values, and a minimum P was regarded as the optimal one in different subgroups.

**Results:**

Large tumor size groups had worse overall survival (OS) than small tumor size groups. Cox regression analysis suggested that tumor size was an independent prognostic factor for OS for solitary ICC after resection. Subgroup analysis showed tumor size was associated with OS for both solitary ICC with and without vascular invasion (VI). Furthermore, the optimal cut-off values for solitary ICC with and without VI were found to be 8 and 3 cm, respectively, which could divide the patients into two groups with significant differences in OS.

**Conclusion:**

Tumor size was an independent prognostic factor for solitary ICC after resection. The existing American Joint Committee on Cancer (AJCC) staging system could be improved if the cut-off value of the T1 stage was changed to 8 cm and if the T2 stage incorporated a tumor size with a cut-off value of 3 cm. Further studies with more cases are needed to validate these findings.

## Introduction

Intrahepatic cholangiocarcinoma (ICC) is the second most prevalent primary liver cancer after hepatocellular carcinoma (HCC), and the incidence of ICC has been increasing worldwide over the last two decades (1, 2). Liver resection is a potential curative strategy that can prolong overall survival (OS) for patients with ICC; however, the prognosis of these patients is still dismal, and recent studies suggest that the 5-year OS of patients with ICC after surgery is only approximately 30% (3). To date, many pathological features have been discovered to be associated with the prognosis of ICC, including vascular invasion (VI), tumor differentiation, tumor size, tumor number, lymph node metastasis, and surgical resection margin (1, 4).

Diameter is a crucial characteristic of solid tumors, and it has been demonstrated to be a prognostic factor for many cancers and integrated into various staging systems to guide treatment and predict prognosis (5). In the 8th American Joint Committee on Cancer (AJCC) cancer TNM staging system for ICC, tumor size is used to differentiate T1 into T1a (≤ 5 cm) and T1b (> 5 cm), while T2 is classified based on VI and tumor number, and T3 and T4 are defined based on the invasion of surrounding tissues or organs (6). The effect of tumor size on survival in ICC has been reported in many studies (7–10); however, the results in these explorations were inconsistent in terms of both the prognostic ability and the identification of optimal cut-off values. The ambiguous status of diameter as a factor in prognosis and the imprecise cut-off point for the diameter could influence the reliability of the existing staging systems and affect the selection of treatment strategy and the prediction of prognosis (7). Worse still, due to the relatively low incidence of ICC, rare studies have focused on the effect of tumor size on survival in patients with solitary ICC after surgery, which makes it difficult to evaluate the predictive efficacy of the existing TNM staging systems for ICC.

In this study, using data obtained from the Surveillance, Epidemiology, and End Results (SEER) Program, we aimed to analyze the clinicopathological characteristics among different tumor size groups and evaluate the prognostic ability of tumor size for solitary ICC after surgery. The optimal cut-off values for different subgroups were explored, and an independent patient cohort from Shandong Provincial Hospital (SDPH) was used to validate the observations in the analysis of the SEER database.

## Materials and Methods

### Ethics Statement

This study was approved by the Ethics Committee of Shandong Provincial Hospital Affiliated to Shandong University and was performed in accordance with the Declaration of Helsinki. Each participant provided written informed consent.

### Patient Selection in SEER Set

**Figure 1:**
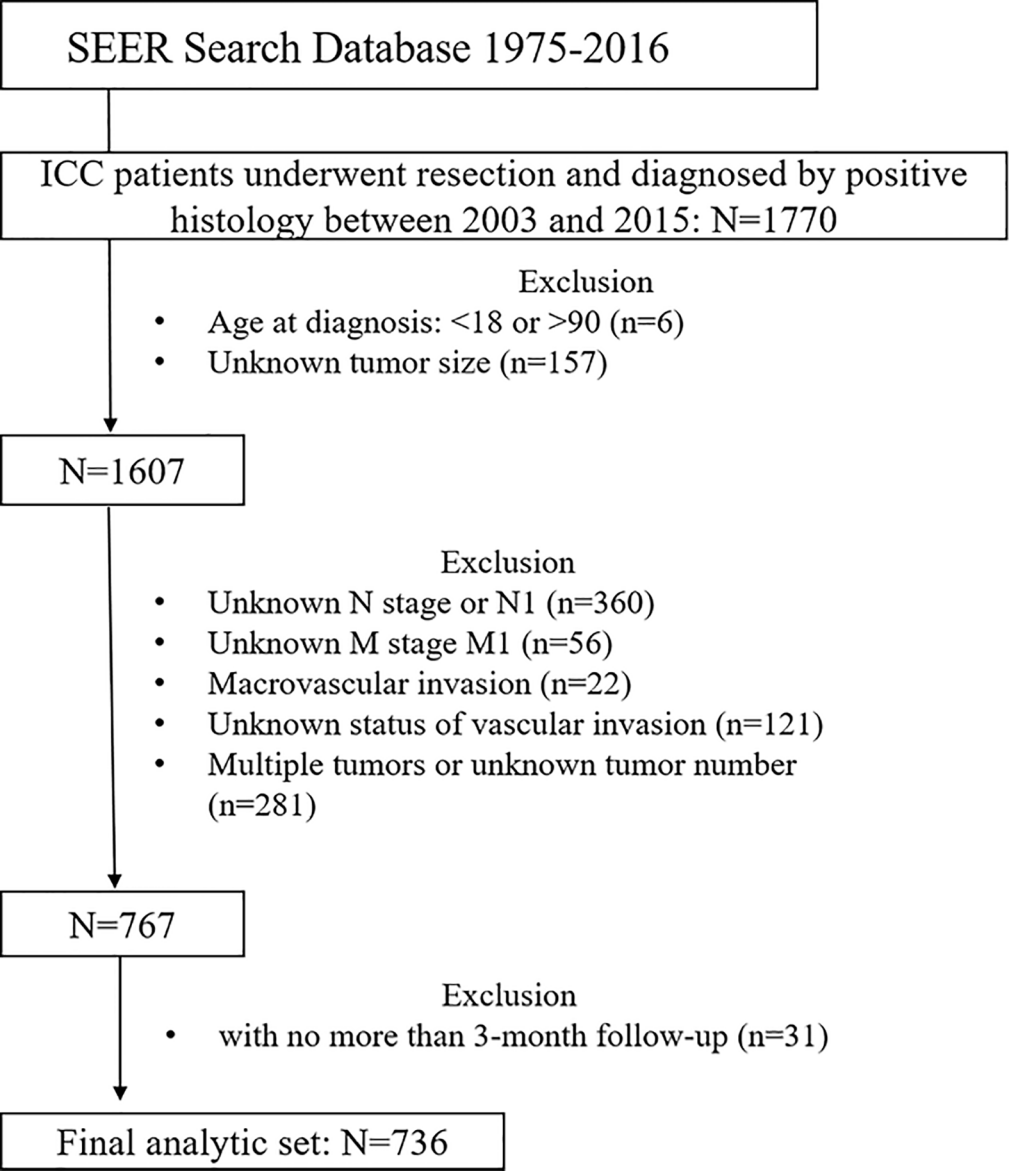
Workflow of patient selection. ICC, intrahepatic cholangiocarcinoma; SEER, The Surveillance, Epidemiology, and End Results Program.

The SEER 18 Regs Research Data (with additional treatment fields, 1975–2016 varying) were used as the data source for this study (https://seer.cancer.gov/), and SEER*Stata software (Version 8.3.5) was employed for analysis. Patients diagnosed between 1975 and 2016 were identified using the cite code C22.0 (liver) and a histological diagnosis of cholangiocarcinoma (International Classification of Diseases for Oncology, 3rd Edition [ICD-O-3] code 8,160) or the cite code C22.1 (intrahepatic bile duct) and histological code 8,140 (adenocarcinoma) or 8,160. Only patients who received surgical treatment and were diagnosed with ICC by positive histology aged between 18 and 90 years were included. Afterwards, patients diagnosed before 2002 were excluded, and those diagnosed in 2016 were also excluded because the follow-up time was less than 12 months. Patients were excluded if they had incomplete clinicopathological information, lymph node or distant metastasis, macrovascular invasion (referred to major branches of portal or hepatic vein, and vena cava), multiple tumors, and incomplete follow-up information or less than 3 months of follow-up time. **Figure 1** shows the workflow of patient selection.

### Patient Selection in the SDPH Set

To validate the findings obtained from the analysis of the SEER program, patients with solitary ICC who received surgical treatment between May 2007 and December 2017 at SDPH were retrospectively analyzed. Patients who had incomplete clinicopathological characteristics, less than 3 months of follow-up time or incomplete follow-up information were excluded. Patients were excluded if they received adjuvant chemotherapy before surgery, were diagnosed at an age of <18 or >90 years old, had lymph node or distant metastasis or had macrovascular invasion. Tumor size was evaluated based on preoperative imaging and recorded according to the greatest dimension of axial images. Patients were postoperatively followed up using regular laboratory tests and imaging examinations at intervals of 2–3 months during the first year after surgery and 3–6 months thereafter. OS was defined as the time from the date of surgery to death or to the latest date of follow-up.

### Statistical Analysis

Tumor size was treated as a categorical variable using cut-off points of 2, 5, and 7 cm, as proposed by the Liver Cancer Study Group of Japan (LCSGJ), the AJCC 8th edition staging system and a multicenter study from Eastern and Western countries (6, 7, 11), and patients were thus classified into four subgroups: Group I (0–2 cm), Group II (2–5 cm), Group III (5–7 cm), and Group IV (>7 cm). Categorical variables are displayed as numbers (n) and proportions (%), and continuous variables are displayed as the median (interquartile range, IQR). The chi-square test and Fisher’s exact test were used to compare variables among different groups. Kaplan-Meier analysis and the log-rank test were used to analyze OS. Moreover, a minimum P value method was used to determine the optimal cut-off points for tumor size, 10 cut-off points ranging from 2 to 11 cm were chosen, and the patients were divided into large and small tumor size groups using different cut-off points. Afterwards, the log-rank test was used to compare the predictive ability of OS of different tumor size thresholds, and a minimum P was regarded as the optimal one (7, 12).

The Cox proportional hazard model was applied to identify independent prognostic factors associated with OS. Variables with p <0.05 in the univariate analysis were regarded as potential risk factors and were included in the multivariate analysis. The hazard ratios (HRs) with 95% confidence intervals (95% CIs) were recorded. All statistical tests were two-sided, and p <0.05 was defined as statistical significance. SPSS version 22.0 (SPSS Inc., Chicago, IL) and R software (version 3.5.2) were employed in the statistical analysis.

## Results

### Demographic and Clinicopathological Characteristics of Patients With ICC

A total of 736 patients in SEER database satisfied the inclusion criteria and were included in the analysis; there were 74 (10.05%) patients in group I, 320 (43.48%) patients in group II, 170 (23.10%) patients in groups III, and 172 (23.37%) patients in group IV. A total of 155 (21.06%) patients were diagnosed with solitary ICC between 2003 and 2007, 234 (31.79%) patients between 2008 and 2011, and 347 (47.15%) patients between 2012 and 2015. Most of the patients (563 patients, 76.49%) were diagnosed between 45 and 75 years old. Three hundred sixty-three (49.32%) patients were male, and 373 (50.68%) patients were female. The median follow-up time was 34 months (IQR: 19 to 61.5 months) for all patients. **Table 1** displayed the patients’ demographic and clinicopathological characteristics.

**Table 1:** Baseline characteristics of patients diagnosed with solitary ICC in SEER database.

Variable	Total (n = 736)	Group 1: 0–2 cm (n = 74)	Group 2: 2–5 cm (n = 320)	Group 3: 5–7 cm (n = 170)	Group 4: >7 cm (n = 172)	P
Year of diagnosis						0.603
2003–2007	155 (21.06)	11 (14.86)	71 (22.19)	34 (20.00)	39 (22.67)	
2008–2011	234 (31.79)	22 (29.73)	100 (31.25)	61 (35.88)	51 (29.65)	
2012–2015	347 (47.15)	41 (55.41)	149 (46.56)	149 (44.12)	82 (47.67)	
Age, years						0.578
18–45	40 (5.43)	4 (5.41)	10 (3.13)	13 (7.65)	13 (7.56)	
46–60	214 (29.08)	22 (29.73)	92 (28.75)	53 (31.18)	47 (27.33)	
61–75	349 (47.42)	36 (48.65)	157 (49.06)	75 (44.12)	81 (47.09)	
>75	133 (18.07)	12 (16.22)	61 (19.06)	29 (17.06)	31 (18.02)	
Sex						0.003
Male	363 (49.32)	49 (66.22)	163 (50.94)	70 (41.18)	81 (47.09)	
Female	373 (50.68)	25 (33.78)	157 (49.06)	100 (58.82)	91 (52.91)	
Race						0.205
White	591 (80.30)	64 (86.49)	256 (80.00)	130 (76.47)	141 (81.98)	
Black	54 (7.34)	1 (1.35)	21 (6.56)	17 (10.00)	15 (8.72)	
Other	91 (12.36)	9 (12.16)	43 (13.44)	23 (13.53)	16 (9.30)	
Insurance						0.351
Uninsured	6 (0.82)	2 (2.70)	1 (0.31)	2 (1.18)	1 (0.58)	
Insured	608 (82.61)	64 (86.49)	262 (81.88)	141 (82.94)	141 (81.98)	
Unknown	122 (16.58)	8 (10.81)	57 (17.81)	27 (15.88)	30 (17.44)	
Tumor differentiation						0.004
Well/Moderate	460 (62.50)	53 (71.62)	209 (65.31)	100 (58.82)	98 (56.98)	
Poor/Undifferentiated	174 (23.64)	5 (6.76)	76 (23.75)	44 (25.88)	49 (28.49)	
Unknown	102 (13.86)	16 (21.62)	35 (10.94)	26 (15.29)	25 (14.53)	
Vascular invasion						0.042
No	545 (74.05)	57 (77.03)	251 (78.44)	121 (71.18)	116 (67.44)	
Yes	191 (25.95)	17 (22.97)	69 (21.56)	49 (28.82)	56 (32.56)	
Chemotherapy						0.025
No/Unknown	502 (68.21)	53 (71.62)	228 (71.25)	120 (70.59)	101 (58.72)	
Yes	234 (31.79)	21 (28.38)	92 (28.75)	50 (29.41)	71 (41.28)	
Follow-up (median, IQR)						
	34 (19–61.5)	33.5 (20–57)	35 (19–65.5)	35 (20–65)	30.5 (18–54)	< 0.001

Data are presented as n (%).

ICC, intrahepatic cholangiocarcinoma; IQR, interquartile range; SEER, Surveillance, Epidemiology, and End Results Program.

### Clinicopathological Characteristics Among Different Tumor Size Groups

As shown in **Table 1**, while the demographic traits among different tumor size groups were comparable, significant differences in clinicopathological characteristics, including sex, tumor differentiation, VI and chemotherapy (yes or No/Unknown), were found. We found that larger tumor size groups had a higher proportion of female patients (p = 0.003), had poorer tumor differentiation (p = 0.004), had a higher proportion of VI (p < 0.001), and had a higher proportion of receiving chemotherapy (p = 0.025).

### Influence of Tumor Size on OS in the SEER Database

**Figure 2:**
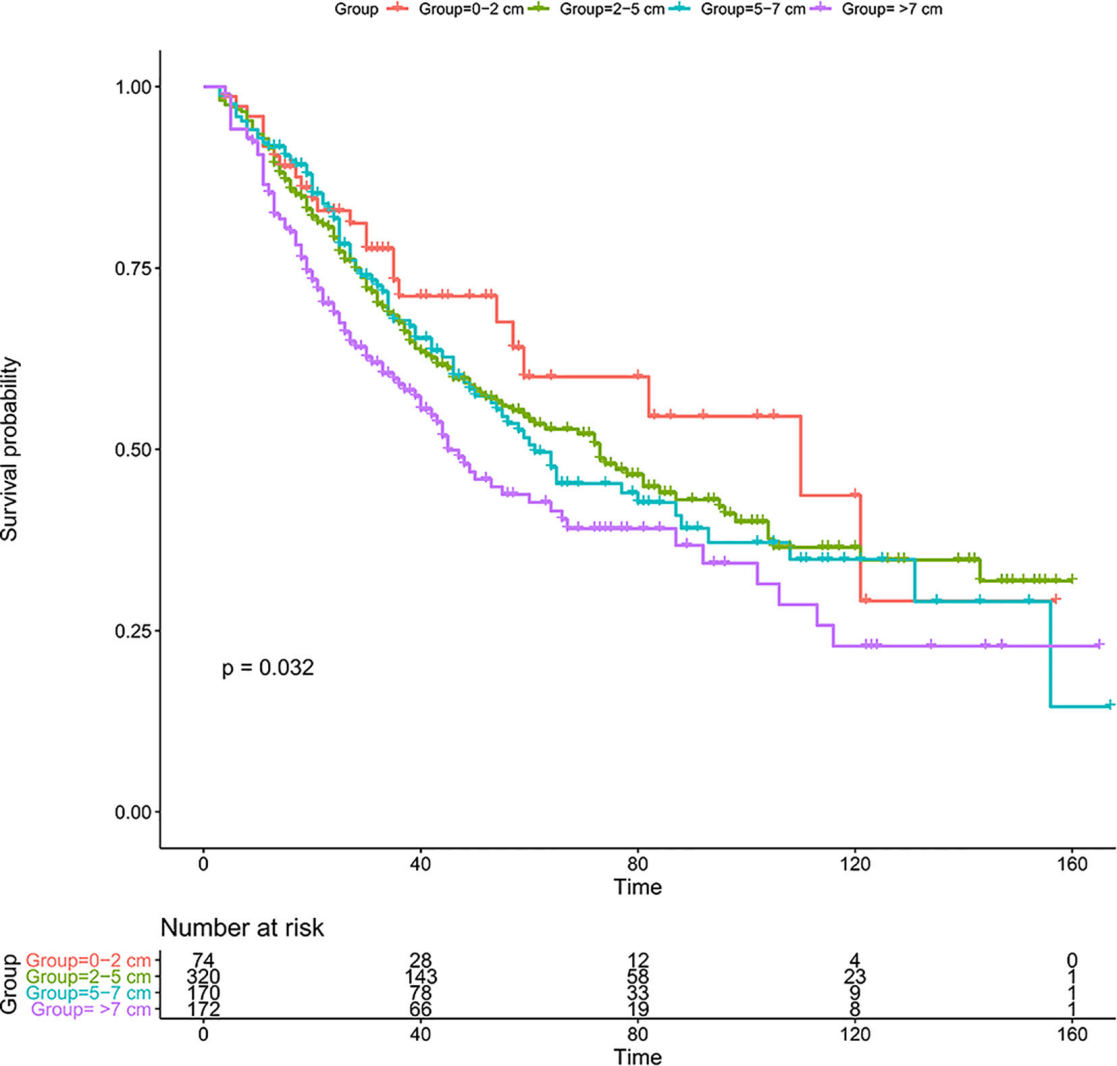
Kaplan-Meier analysis for solitary patients with ICC after resection in the SEER database. ICC, intrahepatic cholangiocarcinoma; SEER, The Surveillance, Epidemiology, and End Results Program.

The 1-, 3-, and 5-year OS was 91.8, 71.1, and 60.0%, respectively, in group I; 91.6, 67.5, and 54.1%, respectively, in group II; 91.7, 67.8, and 50.6%, respectively, in group III; and 85.3, 58.9, and 42.7%, respectively, in group IV. Kaplan-Meier analysis showed that tumor size was associated with the prognosis of solitary ICC after surgery, and larger tumor size groups tended to have a worse prognosis (**Figure 2**, p = 0.032).

Univariate Cox regression analysis showed that age at diagnosis, sex, tumor differentiation, tumor size (treated as a continuous or categorical variable) and VI were associated with the prognosis of solitary ICC (p < 0.05, **Table 2**). Furthermore, multivariate analysis was conducted, and the following 5 variables were independent prognostic factors for solitary ICC after surgery: age at diagnosis (p = 0.014, 46–60: HR 1.689; 95% CI 0.957–2.980; 61–75: HR 1.925; 95% CI 1.106–3.352; >75: HR 2.397; 95% CI 1.341–4.286), sex (female: p = 0.001, HR 1.469, 95% CI 1.183–1.826), tumor differentiation (poor/undifferentiated: p = 0.043, HR 1.384, 95% CI 1.073–1.785), tumor size (continuous variable: p = 0.001, HR 1.006, 95% CI 1.003–1.010; categorical variable: p = 0.044, 2–5 cm: HR 1.257, 95% CI 0.810–1.950; 5–7 cm, HR 1.385, 95% CI 0.870–2.204; >7 cm: HR 1.728, 95% CI 1.094–2.730) and VI (Yes: p = 0.044, HR 1.275, 95% CI 1.006–1.615).

**Table 2:** Analysis of prognostic factors for solitary ICC after resection in SEER database.

Variable	Univariate			Multivariate		
	HR	95%CI	P	HR	95%CI	P
Year of diagnosis						
2003–2007	Reference		0.188			
2008–2011	1.215	0.928–1.590				
2012–2015	0.977	0.719–1.326				
Age, years						
18–45	Reference		0.008	Reference		0.014
46–60	1.757	1.000–3.086		1.689	0.957–2.980	
61–75	2.027	1.169–3.514		1.925	1.106–3.352	
>75	2.509	1.409–4.468		2.397	1.341–4.286	
Sex						
Female	Reference		0.001	Reference		0.001
Male	1.423	1.149–1.763		1.469	1.183–1.826	
Race						
White	Reference		0.811			
Black	1.053	0.713–1.555				
Other	0.913	0.663–1.257				
Insurance						
Uninsured	Reference		0.307			
Insured	0.791	0.197–3.179				
Unknown	0.807	0.611–1.065				
Tumor differentiation						
Well/Moderate	Reference		0.010	Reference		0.043
Poor/Undifferentiated	1.474	1.147–1.892		1.384	1.073–1.785	
Unknown	1.097	0.802–1.499		1.131	0.825–1.549	
Tumor size						
Continuous	1.006	1.002–1.010	0.001	1.006	1.003–1.010	0.001
0–2 cm	Reference		0.035	Reference		0.044
2–5 cm	1.278	0.829–1.969		1.257	0.810–1.950	
5–7 cm	1.321	0.837–2.084		1.385	0.870–2.204	
>7 cm	1.747	1.115–2.738		1.728	1.094–2.730	
Vascular invasion						
No	Reference		0.002	Reference		0.044
Yes	1.430	1.135–1.800		1.275	1.006–1.615	
Chemotherapy						
No/Unknown	Reference		0.259			
Yes	1.140	0.908–1.431				

ICC, intrahepatic cholangiocarcinoma; SEER, Surveillance, Epidemiology, and End Results Program.

### Effect of Tumor Size on OS in Subgroups Classified by VI

A total of 545 and 191 patients were classified into the solitary ICC without VI and with VI subgroups, respectively. The 1-, 3-, and 5-year OS in the different subgroups are displayed in **Table S1**; however, Kaplan-Meier analysis showed that tumor size was not associated with OS in both subgroups (p > 0.05, **Figure S1**).

Furthermore, we conducted Cox regression analysis to explore the prognostic ability of tumor size in different subgroups. As shown in **Table S2**, for solitary ICC without VI, although tumor size was not a prognostic factor when treated as a categorical variable (p = 0.175), it was an independent prognostic factor when regarded as a continuous variable (p < 0.001, HR 1.075, 95% CI 1.040–1.112). For solitary ICC with VI, there was not significant difference for the effect of tumor size on OS when it was treated as categorical variable (p < 0.001); however, when it was treated as a continuous variable, tumor size had an influence on OS (p = 0.055, HR 1.005, 95% CI 1.000–1.011).

### Identification of Optimal Cut-Off Values in Subgroups Classified by VI

**Figure 3:**
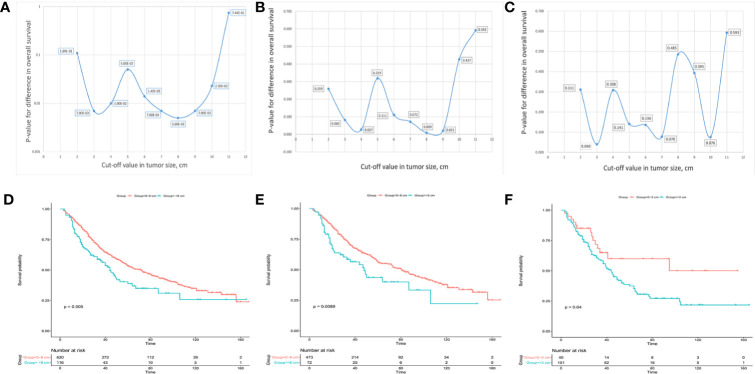
Charts for different cut-off points with corresponding p values and Kaplan-Meier analysis for the optimal cut-off points in solitary ICC **(A, D)**, solitary ICC without VI **(B, E)** and solitary ICC with VI **(C, F)**.

Afterwards, 10 cut-off points ranging from 2 to 11 cm were chosen, and the log-rank test was used to determine the optimal cut-off values in different groups. For solitary ICC, an optimal cut-off value of tumor size of 8 cm could divide the patients into two subgroups with 1-, 3-, and 5-year OS rates of 91.4, 67.5, and 53.3%, respectively, for 0 to 8 cm tumors and 83.5, 57.5, and 38.7%, respectively, for >8 cm tumors (**Figures 3A, C**). For solitary ICC without VI, an optimal cut-off value of 8 cm could divide the patients into two subgroups with 1-, 3-, and 5-year OS rates of 92.4, 69.8, and 56.3%, respectively, for 0 to 8 cm tumors, and 86.1, 56.4, and 43.6%, respectively, for >8 cm tumors (**Figures 3B, D**). For solitary ICC with VI, an optimal cut-off value of 3 cm could divide the patients into two subgroups with 1-, 3-, and 5-year OS rates of 90.0, 65.0, and 60.0%, respectively, for 0 to 3 cm tumors and 85.4, 58.4, and 37.5%, respectively, for >3 cm tumors (**Figures 3C, E**). The ROC curves also suggested that the three cut-off values had predictive efficiencies for ICC prognosis (**Figure S2**).

### Validating the Prognostic Ability of Tumor Size in the SDPH Set

To validate the findings in the SEER set, a total of 129 eligible patients with solitary ICC from the SDPH set were analyzed as the external validation dataset. The demographic and clinicopathological characteristics of the patients are shown in **Table 3**.

**Table 3:** Clinicopathologic Features of Patients with ICC in the SDPH set.

Variable	N (n = 129)	%
Age, median (IQR)	59 (52–66)	
Sex		
Male	69	53.49
Female	60	46.51
GGT, U/L		
≤54	63	48.84
>54	66	51.16
CA199, U/ml		
≤37	50	38.76
>37	79	61.24
CEA, ng/ml		
≤5	102	79.07
>5	27	20.93
Tumor size, cm		
Median (IQR)	5.1 (3.6–7.2)	
0–2	8	6.20
2–5	55	42.64
5–7	33	25.58
≥7	33	25.58
Vascular invasion		
No	95	73.64
Yes	34	26.36
Tumor differentiation		
Poor	23	17.83
Moderate/High	106	82.17

ICC, intrahepatic cholangiocarcinoma; SDPH, Shandong Provincial Hospital; GGT, γ-glutamyl transferase; CA19-9, carbohydrate antigen 19-9; CEA, carcinoembryonic antigen; IQR, interquartile range.

**Figure 4:**
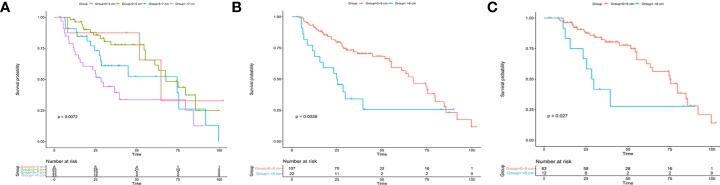
Kaplan-Meier analysis for patients with solitary ICC after resection in the SDPH set. **(A)** Solitary ICC with cut-off values of 2, 5, and 7 cm; **(B)** solitary ICC with a cut-off value 8 cm; **(C)** solitary ICC without VI with a cut-off value of 8 cm. ICC, intrahepatic cholangiocarcinoma; SDPH, Shandong Provincial Hospital; VI, vascular invasion.

We first studied the prognostic ability of tumor size in the SDPH set. Kaplan-Meier analysis showed that tumor size was associated with OS for patients with ICC (p = 0.0072, **Figure 4A**). Moreover, Cox analysis suggested that tumor size was an independent prognostic factor for OS when it was treated as a continuous variable (p = 0.031, HR 1.116, 95% CI 1.010–1.234), while no significant difference was found when it was treated as a categorical variable (p = 0.090) (**Table 4**). Afterwards, we tested the optimal cut-off values in the SDPH set. Kaplan-Meier analysis showed that patients with 0–8 cm tumors had better OS than those with >8 cm tumors in the solitary ICC (p = 0.0028, **Figure 4B**) and solitary ICC without VI subgroups (p = 0.027, **Figure 4C**). In the Cox regression analysis, we discovered that tumor size was an independent prognostic factor for OS in the solitary ICC (p = 0.015, HR 2.115, 95% CI 1.153–3.879) and solitary ICC without VI subgroups (p = 0.032, HR 2.369, 95% CI 1.075–5.221) when it was treated as a categorical variable, and the cut-off value was 8 cm (**Table S3**). However, for solitary ICC with VI, since there were only three patients with a tumor size ≤3 cm, we failed to evaluate the efficacy of cut-off value of 3 cm for the prediction of OS.

**Table 4:** Analysis of prognostic factors for ICC in the SDPH set.

Variable	Univariate			Multivariate		
	HR	95%CI	P	HR	95%CI	P
Age, years						
18–45	Reference		0.961			
46–60	0.882	0.309–2.518				
61–75	0.795	0.277–2.284				
>75	0.704	0.078–6.357				
Sex						
Male	Reference		0.989			
Female	1.004	0.606–1.662				
GGT, U/L						
≤54	Reference		0.001	Reference		0.007
>54	2.444	1.438–4.153		2.128	1.233–3.672	
CA19-9, U/ml						
≤37	Reference		0.038	Reference		0.651
>37	1.795	1.033–3.119		1.143	0.640–2.043	
CEA, ng/ml						
≤5	Reference		0.552			
>5	1.206	0.650–2.236				
Tumor differentiation						
Moderate/High	Reference		0.980			
Poor	1.009	0.517–1.967				
Tumor size						
Continuous	1.164	1.062–1.277	0.001	1.116	1.010–1.234	0.031
0–2 cm	Reference		0.010	Reference		0.090
2–5 cm	1.107	0.325–3.777		0.784	0.223–2.758	
5–7 cm	1.857	0.537–6.422		0.997	0.277–3.588	
>7 cm	3.011	0.882–10.275		1.732	0.487–6.161	
Vascular invasion						
No	Reference		<0.001	Reference		<0.001
Yes	3.651	2.096–6.361		3.583	2.009–6.391	

ICC, intrahepatic cholangiocarcinoma; SDPH, Shandong Provincial Hospital; GGT, γ-glutamyl transferase; CA19-9, carbohydrate antigen 19-9; CEA, carcinoembryonic antigen.

## Discussion

Although it has been integrated into the latest AJCC staging system, the roles of tumor size in ICC prognosis, both its prognostic ability and optimal cut-off value, are still controversial (7, 10, 11, 13). In this study, using data collected from the SEER registry, we classified solitary ICC into 4 groups based on tumor size and found that a larger tumor size was associated with various malignant variables. Afterwards, we discovered that larger tumor size groups were associated with poorer OS and that tumor size was an independent prognostic factor for solitary ICC. We further explore the prognostic ability of tumor size in different subgroups stratified by VI, and Cox regression analysis showed it was an independent prognostic factor. The log-rank test found that 8, 8 and 3 cm were the optimal cut-off values for solitary ICC, solitary ICC without VI and solitary ICC with VI, respectively. Finally, a patient cohort containing 129 cases from our center was used to validate the findings in the analysis of the SEER program, and tumor size gave similar results.

Diameter is an important characteristic of solid tumors and has been shown to be associated with OS in various solid cancers, such as hepatocellular carcinoma and non-small cell lung cancer (14, 15). The prognostic ability of tumor size has also been discussed widely in ICC. In a previous population-based cohort study, Nathan et al. (10) retrospectively analyzed 598 patients with ICC from SEER database between 1998 and 2004, proposed a staging system and proved that tumor size was not a prognostic factor for OS. In evaluating the predictive ability of the AJCC 7th edition staging system, Li et al. (16) reported that tumor size was not associated with OS and RFS for patients with ICC. Several studies also suggested that tumor size might not influence the prognosis of patients with ICC after surgery (17–19). As a result, tumor size was not integrated into the previous AJCC staging systems (6). However, most recently, the prognostic ability of tumor size for OS has been demonstrated in several large cohort studies (20). In a multicenter study containing 514 patients from Eastern and Western countries, Hyder et al. (11) demonstrated that tumor size was an independent prognostic factor for OS in ICC and constructed a prognostic nomogram. Wang et al. (4) also obtained similar conclusions in a study containing 367 patients with ICC. Consequently, tumor size was included in the latest AJCC staging system and was used to predict prognosis for patients with ICC. However, the optimal cut-off value for tumor size to predict prognosis is still controversial (6, 7, 11), and since rare studies have focused on the prognostic ability of tumor size in solitary ICC, it is difficult to conclude more concrete results for these issues.

In our study, by retrospectively analyzing patients with solitary ICC from the SEER database between 2003 and 2015, we found that tumor size was an independent prognostic factor for solitary ICC after surgery. We then further explored the prognostic ability of tumor size in different subgroups classified by VI. Consistent with the AJCC TNM staging system, for solitary ICC without VI, we found that tumor size was associated with OS; however, we discovered that 8 cm was the optimal cut-off value instead of 5 cm. Interestingly, many other studies have also proposed a larger than 5 cm cut-off point for evaluating the prognostic ability of tumor size for ICC. For instance, in a multi-institutional database study, Spolverato et al. (21) proved that patients with ICC with a tumor size >7 cm had poorer recurrence-free survival and OS, which was similar to the findings of Sahara’s studies (11, 22). Hyder et al. (11) also found that 7 cm was the best cut-off point to assess the effect of diameter on the HR of mortality. For patients with solitary ICC with VI who were classified in the T2 stage according to the latest AJCC staging system, we also found that tumor size was a prognostic factor and that 3 cm was the optimal cut-off value. However, due to the limited number of study cases, we failed to evaluate this finding in the external set. A small cut-off value for ICC has also been discussed in several previous studies (7, 8). In a recent study, Ruzzenente et al. (23) found that patients with ICC with tumors ≤3 cm had better OS than those with a larger tumor size. Based on our findings, for the AJCC TNM staging system, we proposed that there should be a change in the cut-off value for T1, tumor size should be incorporated into T2 stage, and 3 cm could be an appropriate cut-off value.

Our results also suggested that solitary ICC was a heterogeneous group, and larger tumor size was associated with malignant pathological factors, including worse tumor differentiation (20, 22) and VI (20, 24). It was believed that a larger tumor size was associated with various malignant variables (25–28). The prognostic ability of these variables has also been reported in ICC (4, 11, 22), and tumor size has been integrated into several risk systems to predict the presence of malignant pathological factors and OS (24, 29, 30). However, many studies have reported that the negative effect of tumor size on OS was largely due to the association between tumor size and malignant variables instead of tumor size itself (10, 31). In this study, using multivariate Cox regression analysis, we still found that tumor size was an independent prognostic factor for solitary ICC. Based on these findings, we hypothesized that tumor size reflected ICC progression and might be used to predict the presence of certain malignant pathological factors and OS.

This study was performed using a large number of patients with solitary ICC, and an independent external patient cohort was employed for the validation of related findings. However, limitations still exist. First, this was a retrospective study, and the findings should be further validated in other institutions with more patients in a prospective style. Second, due to the small number of patients in external set, we failed to evaluate 3 cm as the optimal cut-off value for solitary ICC with VI; thus, further studies with more patients should be conducted to evaluate this issue. Third, several crucial prognostic factors, such as carbohydrate antigen 19-9 (CA19-9) levels, postoperative complications and treatment strategy (R resection, surgical options), were unavailable in SEER database, which could influence the analysis in our study.

## Conclusions

In conclusion, this study proved that tumor size was associated with several malignant clinicopathological features and was an independent prognostic factor for solitary ICC after resection. The current 8th AJCC staging system might be improved by changing the cut-off value in T1 stage from 5 to 8 cm and incorporating tumor size into the T2 stage with a cut-off value of 3 cm.

## Data Availability Statement

The raw data supporting the conclusions of this article will be made available by the authors, without undue reservation.

## Ethics Statement

The studies involving human participants were reviewed and approved by the Ethics Committee of Shandong Provincial Hospital Affiliated to Shandong University. The patients/participants provided their written informed consent to participate in this study.

## Author Contributions

JK, JL, and JW contributed to the conception and design of the study. XL, YC, and JC collected patients’ details and extracted data. XL, CL, and JC analyzed the data. JK and YC drafted the manuscript. JL, JW, and CL contributed with a critical revision of the manuscript. All authors contributed to the article and approved the submitted version.

## Funding

This study was supported by the National Natural Science Foundation of China (No. 81373172, 81770646) and Shandong Provincial Natural Science Foundation, China (No. ZR2014HP065).

## Conflict of Interest

The authors declare that the research was conducted in the absence of any commercial or financial relationships that could be construed as a potential conflict of interest.

## Supplementary Material

Supplementary Figure 1 Kaplan-Meier analysis for solitary ICC after resection in different subgroups from the SEER database. **(A)** Solitary ICC without VI; **(B)** solitary ICC with VI. ICC, intrahepatic cholangiocarcinoma; SEER, The Surveillance, Epidemiology, and End Results Program; VI, vascular invasion.

Supplementary Figure 2 ROC curves analysis for the predictive efficiency of tumor size for ICC prognosis at 1, 3, and 5 years in different subgroups. **(A)** Solitary ICC; **(B)** Solitary ICC without VI; **(C)** solitary ICC with VI. ICC, intrahepatic cholangiocarcinoma; SEER, The Surveillance, Epidemiology, and End Results Program; VI, vascular invasion.
