# Supplementary figures and images for: Effect of Tumor Size on Long-Term Survival After Resection for Solitary Intrahepatic Cholangiocarcinoma
Source: Front Oncol. 2021 Jan 21;10:559911. doi: 10.3389/fonc.2020.559911 (PMC7859518; doi:10.3389/fonc.2020.559911)

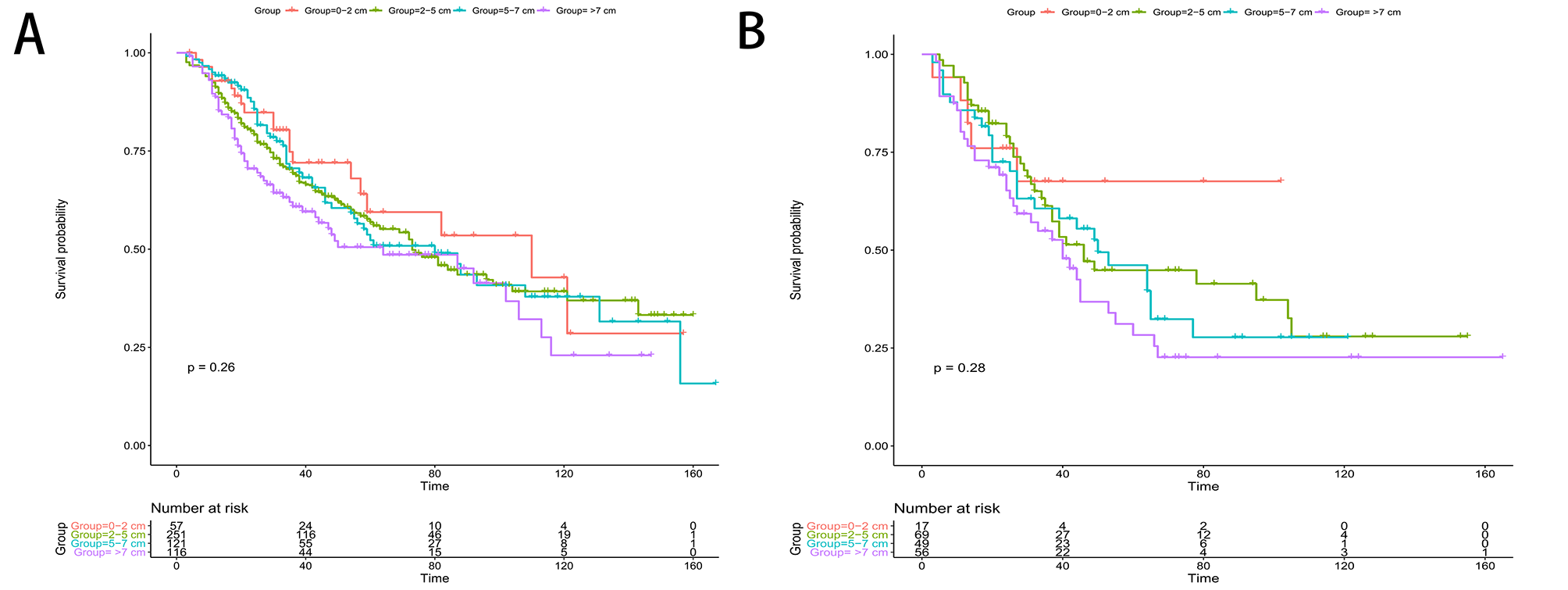

Supplement: Supplementary Figure 1 — Kaplan-Meier analysis for solitary ICC after resection in different subgroups from the SEER database. (A) Solitary ICC without VI; (B) solitary ICC with VI. ICC, intrahepatic cholangiocarcinoma; SEER, The Surveillance, Epidemiology, and End Results Program; VI, vascular invasion. [file Image_1.tif]

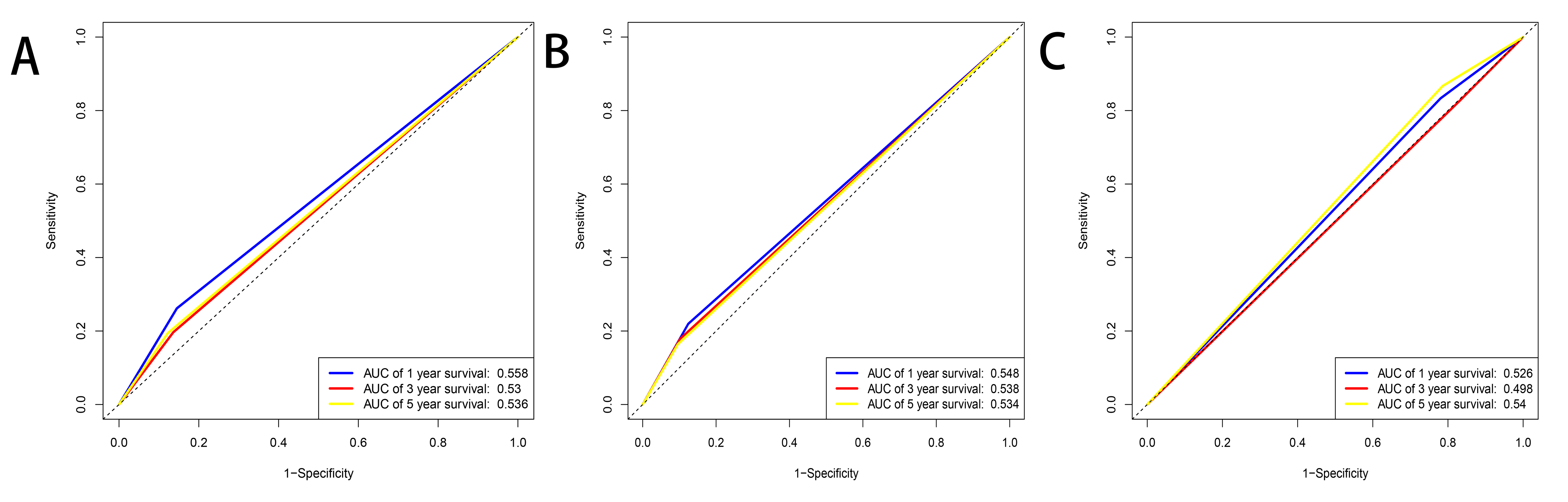

Supplement: Supplementary Figure 2 — ROC curves analysis for the predictive efficiency of tumor size for ICC prognosis at 1, 3, and 5 years in different subgroups. (A) Solitary ICC; (B) Solitary ICC without VI; (C) solitary ICC with VI. ICC, intrahepatic cholangiocarcinoma; SEER, The Surveillance, Epidemiology, and End Results Program; VI, vascular invasion. [file Image_2.tif]
