# Supplementary material for: Effect of Tumor Size on Long-Term Survival After Resection for Solitary Intrahepatic Cholangiocarcinoma
Source: Front Oncol. 2021 Jan 21;10:559911. doi: 10.3389/fonc.2020.559911 (PMC7859518; doi:10.3389/fonc.2020.559911)
Supplement: Supplementary file 3 [file Table_1.docx]

Table S1. The 1-, 3- and 5-year OS in different subgroups of the SEER database

| Year | Tumor size, cm | Subgroup | |
| --- | --- | --- | --- |
|  |  | Solitary ICC without VI | Solitary ICC with VI |
| 1 | 0-2 | 92.9 % | 88.2 % |
|  | 2-5 | 91.2 % | 92.8 % |
|  | 5-7 | 94.2 % | 85.7 % |
|  | >7 | 88.7 % | 78.4 % |
| 3 | 0-2 | 72.0 % | 67.6 % |
|  | 2-5 | 69.3 % | 61.3 % |
|  | 5-7 | 70.6 % | 60.6 % |
|  | >7 | 60.8 % | 54.9 % |
| 5 | 0-2 | 59.4 % | 67.6 % |
|  | 2-5 | 56.8 % | 44.9 % |
|  | 5-7 | 52.3 % | 46.2 % |
|  | >7 | 50.5 % | 28.3 % |

Abbreviations: OS, overall survival; SEER, Surveillance, Epidemiology, and End Results Program; VI, vascular invasion.
