# Supplementary material for: Effect of Tumor Size on Long-Term Survival After Resection for Solitary Intrahepatic Cholangiocarcinoma
Source: Front Oncol. 2021 Jan 21;10:559911. doi: 10.3389/fonc.2020.559911 (PMC7859518; doi:10.3389/fonc.2020.559911)
Supplement: Supplementary file 4 [file Table_2.docx]

Table S2. Multivariate Cox regression analysis of prognostic factors for solitary ICC without or with VI in the SEER database

| Variable | Solitary ICC without VI | | | Solitary ICC with VI | | |
| --- | --- | --- | --- | --- | --- | --- |
|  | HR | 95%CI | P | HR | 95%CI | P |
| Age |  |  |  |  |  |  |
| 18-45 | Reference |  | 0.013 |  |  |  |
| 46-60 | 1.811 | 0.973-3.372 |  |  |  |  |
| 61-75 | 1.925 | 1.046-3.542 |  |  |  |  |
| >75 | 2.672 | 1.413-5.055 |  |  |  |  |
| Sex |  |  |  |  |  |  |
| Male | Reference |  | <0.001 |  |  |  |
| Female | 0.591 | 0.453-0.770 |  |  |  |  |
| Tumor differentiation | |  |  |  |  |  |
| Well/Moderate | Reference |  |  |  |  |  |
| Poor/ Undifferentiated | 1.393 | 1.007-1.927 | 0.045 |  |  |  |
| Unknown | 1.259 | 0.884-1.792 | 0.201 |  |  |  |
| Tumor size |  |  |  |  |  |  |
| Continuous | 1.075 | 1.040-1.112 | <0.001 | 1.005 | 1.000-1.011 | 0.055 |
| 0-2 cm | Reference |  | 0.175 | Reference |  | 0.298 |
| 2-5 cm | 1.283 | 0.779-2.111 | 0.327 | 1.356 | 0.531-3.461 | 0.525 |
| 5-7 cm | 1.397 | 0.815-2.395 | 0.225 | 1.511 | 0.582-3.922 | 0.396 |
| >7 cm | 1.713 | 1.008-2.911 | 0.047 | 1.907 | 0.772-5.027 | 0.156 |

Abbreviations: ICC, intrahepatic cholangiocarcinoma; VI, vascular invasion; SEER, Surveillance, Epidemiology, and End Results Program.
