# Supplementary material for: Effect of Tumor Size on Long-Term Survival After Resection for Solitary Intrahepatic Cholangiocarcinoma
Source: Front Oncol. 2021 Jan 21;10:559911. doi: 10.3389/fonc.2020.559911 (PMC7859518; doi:10.3389/fonc.2020.559911)
Supplement: Supplementary file 5 [file Table_3.docx]

Table S3. Multivariate Cox regression analysis of prognostic factors for solitary ICC without or with VI in the SDPH set

|  | Solitary ICC | | | Solitary ICC without VI | | |
| --- | --- | --- | --- | --- | --- | --- |
|  | HR | 95%CI | P | HR | 95%CI | P |
| Tumor size |  |  |  |  |  |  |
| 0-8 cm | Reference |  | 0.015 |  |  | 0.032 |
| >8 cm | 2.115 | 1.153-3.879 |  | 2.369 | 1.075-5.221 |  |

Abbreviations: ICC, intrahepatic cholangiocarcinoma; VI, vascular invasion; SDPH, Shandong Provincial Hospital.
